# The Use of Genotoxicity Endpoints as Biomarkers of Low Dose Radiation Exposure in Interventional Cardiology

**DOI:** 10.3389/fpubh.2021.701878

**Published:** 2021-07-23

**Authors:** Martha Habibi, Panagiotis K. Karyofyllis, Aggeliki Nikolakopoulou, Panagiotis Papagiannis, Pantelis Karaiskos, Alexandros G. Georgakilas, Vasiliki I. Hatzi, Ioannis Malakos, Nikolaos Kollaros, Irene Mastorakou, Vassilis Voudris, Georgia I. Terzoudi

**Affiliations:** ^1^Laboratory of Health Physics, Radiobiology & Cytogenetics, Institute of Nuclear & Radiological Sciences & Technology, Energy & Safety (INRASTES), National Centre for Scientific Research “Demokritos”, Athens, Greece; ^2^Medical Physics Laboratory, Medical School, National and Kapodistrian University of Athens, Athens, Greece; ^3^Division of Interventional Cardiology, Onassis Cardiac Surgery Center, Athens, Greece; ^4^DNA Damage Laboratory, Department of Physics, School of Applied Mathematical and Physical Sciences, National Technical University of Athens (NTUA), Athens, Greece; ^5^Imaging Department, Onassis Cardiac Surgery Center, Athens, Greece

**Keywords:** γ-H2AX foci, chromosomal aberrations, cardiac interventional procedures, low dose radiation effects, micronuclei

## Abstract

The effect of the reportedly low ionizing radiation doses, such as those very often delivered to patients in interventional cardiology, remains ambiguous. As interventional cardiac procedures may have a significant impact on total collective effective dose, there are radiation protection concerns for patients and physicians regarding potential late health effects. Given that very low doses (<100 mSv) are expected to be delivered during these procedures, the purpose of this study was to assess the potency and suitability of current genotoxicity biomarkers to detect and quantitate biological effects essential for risk estimation in interventional cardiology. Specifically, the biomarkers γ-H2AX foci, dicentric chromosomes, and micronuclei, which underpin radiation-induced DNA damage, were studied in blood lymphocytes of 25 adult patients before and after interventional cardiac procedures. Even though the mean values of all patients as a group for all three endpoints tested show increased yields relative to baseline following medical exposure, our results demonstrate that only the γ-H2AX biomarker enables detection of statistically significant differences at the individual level (*p* < 0.001) for almost all patients (91%). Furthermore, 24 h after exposure, residual γ-H2AX foci were still detectable in irradiated lymphocytes. Their decline was found to vary significantly among the individuals and the repair kinetics of γ-H2AX foci was found to range from 25 to 95.6% of their maximum values obtained.

## Introduction

The extensive use of low doses of ionizing radiation ( ≤ 100 mSv) for diagnostic and therapeutic purposes increases concern on the radiation safety of both patients and physicians (1, 2). Ionizing radiation (IR) exposures have been significantly increased during the last decade (3), mainly due to the rise in medical diagnostic and therapeutic interventions, which are responsible for ~40% of the cumulative effective dose of radiation to the population (4, 5). Despite the wide use of low-ionizing-radiation doses and the recent evidence that cancer risk may increase even at lower doses (50–100 mSv) (4, 6), the effects of such exposures in patients exposed to cardiac images and interventional cardiology procedures remain unclear. According to epidemiologic literature, the impact of low doses is hampered by limited statistical power at radiation levels of <100 millisieverts (mSv), even for very large studies (7, 8).

The IR induced DNA damage includes double and single-strand breaks DSBs, base damage BD and DNA protein crosslinks. Among the induced biological effects, DSBs as a genuine type of clustered lesions, are considered the most cytotoxic and carcinogenic. DSBs can be repaired giving apparently normal chromosomes in daughter cells (9) that may promote genomic instability (10). There are several biological endpoints applied for genotoxicity studies and biomonitoring purposes. Among these, the phosphorylation of the H2AX histone to form γ-H2AX foci has been shown to be an accurate biomarker of IR exposure, especially at low doses (11, 12). So, the induction and repair processes of DSBs can be visualized and quantified by using the highly sensitive epigenetic biomarker γ-H2AX, a phosphorylated histone H2A variant (12). Especially, studies have shown that the immunofluorescence analysis of the γ-H2AX foci in peripheral blood lymphocytes is a very sensitive method to visualize DSBs after medical radiographic examinations (11, 13) and very low doses. The γ-H2AX method is a more recent method for radiation dose assessment as compared to earlier established methods like the dicentric chromosome analysis, the cytokinesis-block micronucleus assay (CBMN) and the FISH-translocation assay (14).

In the present study we evaluate the potency and suitability of current genotoxicity biomarkers in peripheral blood lymphocytes to detect and quantitate biological effects, which may be proved critical for risk estimation in interventional cardiology. Lymphocytes are advantageous for exposure assessments because they circulate throughout the body and are continuously exchanged with lymphocytes in tissues. This means that lymphocytes with chromosome aberrations that have been induced anywhere in the body will eventually be present in the peripheral blood (15). Specifically, the biomarkers γ-H2AX foci, dicentric chromosomes, and micronuclei, which underpin radiation-induced complex DNA damage, usually misrepaired or not repaired at all, were studied in 25 adult patients before and after interventional cardiac procedures. Previous studies (16, 17) have shown that chromosomal aberrations and micronuclei (MN) frequencies detected in peripheral blood lymphocytes are directly linked to damage caused by IR and are both crucial predictors of the degree of radiation damage. From one side, it is reported that among the biological dosimetry assays applied in radiation emergency medicine, conventional chromosome analysis using Giemsa-staining to detect dicentric and ring chromosomes has been established as the gold standard for biological dosimetry (18). Studies also report that chromosomal abnormalities such as dicentrics and rings can be detected following chronic or low-dose radiation exposure (19). From the other side, the key advantages of the micronucleus assay lie in its ability to detect both clastogenic and aneugenic events (20). However, an overall estimation of IR risk at very low doses is complicated and depends on the category and dose of radiation, irradiation conditions, body and tissue radiosensitivity, all of which hugely impact the degree of damage and potential late health effects.

## Materials and Methods

### Patients and Blood Sampling

Medical history was obtained from every patient, including demographic data and anthropomorphic variables [weight, height, body mass index (BMI)].

Blood samples (6–7 ml) from 25 patients who underwent ordinary interventional cardiology procedures, such as Coronary Angiography (CA), Percutaneous Transluminal Coronary Angioplasty (PTCA), and ablation were collected directly before, and immediately after the end of the procedure and incubated in heparin-containing vials for two time intervals (0 and 24 h). The blood were stored on ice to inhibit DNA repair (21) during their transfer from the hospital to the laboratory where they were incubated at 37°C for about 20 min for either cell culture initiation or cell lymphocyte isolation and fixation, depending on the assay performed.

Moreover, blood samples of each patient obtained before the cardiac procedures were transferred to the laboratory where they immediately irradiated *in vitro* with 1 Gy of γ-rays (Co-60 Gamma Cell 220 irradiator, Atomic Energy of Canada Ltd., Ottawa, Canada) at the National Center for Scientific Research (NCSR) “Demokritos,” to be used as a positive control. After the *in vitro* irradiation, the experimental procedures were performed according to the detailed protocols described in the next paragraphs. Written informed consent was obtained as the project involves the use of human genetic material and biological samples.

INCLUSION CRITERIA: Patients older than 18 years old scheduled for interventional cardiology procedures (CA, PTCA, and ablation).

EXCLUSION CRITERIA:

1. Patients with an acute myocardial infarction and primary PTCA
2. Patients with a history of cancer treated with radiation therapy or receiving chemotherapeutics
3. Patients who underwent myocardial scintigraphy within the last month
4. Patients who underwent computed tomography within the last month
5. Patients with a PTCA, ablation, or CA, within the last month
6. Patients with a history of leukemia or lymphoma.

### γ-H2AX Foci Analysis for Estimation of the DSBs and Repair

For γ-H2AX foci analysis peripheral blood samples were kept for 20 min at 37°C and then lymphocytes are isolated using Biocoll Separating Solution (1:2) (22) following standard procedures. The cells are kept at 37°C for two time intervals (0 and 24 h). Lymphocytes were washed with a mammalian cell culture medium (RPMI 1640), centrifuged at 1,400 rpm for 10 min and washed with a hypotonic KCl solution (75 mM). An appropriate amount of the cell pellet was placed on microscope slides by means of a cytospin centrifuge at 800 rpm for 4 min. The fixation process and the immunostaining were performed according to the protocol described by Rogakou et al. (12). Afterwards, indirect immunofluorescence assay was performed. The main steps were the permeabilization of the cells, blocking of non-specific binding, immunostaining with primary γ-H2AX antibody (rabbit 1:1,000, Cat: NB100-79967, Novus Biologicals, Abingdon, UK) and secondary fluorescent antibody (Rhodamine Red-X anti-rabbit, 1:4,000, Cat: R6394, Life Technologies). The slides were dried and DAPI gel mount (2%) was added to the cells, then covered with coverslips and stored in the dark prior to analysis under a fluorescent microscope (Axioplan 2, Carl Zeiss Microscopy GmbH, Hamburg, Germany), using the Isis imaging software by Metasystems, Altlussheim, Germany. The number of foci in 200 nuclei were analyzed for each experimental point.

### Peripheral Blood Culture for Dicentric Chromosomal Analysis

For dicentric chromosome analysis, cells were cultured in RPMI 1640 medium containing 10% fetal bovine serum, 1% glutamine, and antibiotics [penicillin: 10,000 U/ml; streptomycin: 10,000 μg/ml (Sigma-Aldrich)]. Phytohaemagglutinin (PHA) was dissolved in water at a concentration of 0.24 mg/ml. Cultures were incubated at 37°C in a humidified incubator in an atmosphere of 5% CO_2_ and 95% air for 48 h. Colcemid solution was added 3 h before cell harvest, and cells were collected by centrifugation, treated in 75 mM KCl for 10 min, fixed in methanol: glacial acetic acid 3:1 (v/v) and processed for cytogenetic analysis. Giemsa staining was achieved by immersing slides in 2% Giemsa solution for 10 min, then washed with distilled water and air dried. Slides were covered with coverslips and analyzed using a microscope (Axioplan 2, Carl Zeiss Microscopy GmbH, Hamburg, Germany). The chromosome aberration analysis, was greatly facilitated by the use of the specific software IKAROS by the semi-automated image analysis system (MetaSystems, Germany). The number of metaphases analyzed was 1,000 for each experimental time point.

### Cytokinesis-Block Micronucleus Assay (CBMN Test)

The cytokinesis-block micronucleus assay (CBMN test) is a robust quantitative assay of chromosome damage by developing the cytokinesis-block technique. In this technique cytochalasin-B (Cyt-B), is added to cell cultures, an inhibitor of the mitotic spindle that prevents cytokinesis. As a consequence, cells that have completed one nuclear division are identified by their binucleated appearance.

The peripheral blood samples obtained were cultured at 37°C with RPMI 1640 medium for 26 h. Cytochalasin-B (Sigma, St. Louis, MO; final concentration, 5.56 μg/mL) has been added and samples were incubated for another 46 h. Cells were then harvested and fixed according to the standard methods (23) and stained for 12 min in 5% Giemsa. For each sample, 1,000 binucleated cells were analyzed using optical microscopy for micronuclei (MN).

### Determination of Mean Effective Dose to Each Patient per Examination

The most commonly available measurement of patient's exposure to IR is the dose area product (DAP), since all modern angiographs are equipped with a DAP meter. The physical quantity DAP quantifies IR output, combined with the irradiated area, and provides a patient dose measure (24). The risk of inducing a radiogenic malignancy from a given X-ray examination can be calculated using the absorbed dose delivered to each exposed organ in the body weighted by those organs' radiosensitivity. Multiplying DAP by a conversion factor is the method of choice for calculating effective dose (ED) [in millisievert (mSv)] in several studies. In this study, a conversion factor of 0.26 mSv/Gycm^2^ was used (25), which has been shown to be more relevant to current practice as it is calculated for higher filtration, routinely implemented in modern systems.

### Statistical Analysis

The statistical analysis of the results was performed as follows: Continuous variables were expressed as the mean ± standard deviation (SD), and the categorical variables were expressed as counts and percentages. Continuous variables were compared using a Student's *t*-test or paired *t*-test for normally distributed value. All tests were considered to be significant at a 0.05 level of statistical significance. Evaluation of normal distribution was performed using the Shapiro-Wilk and Kolmogorov-Smirnov tests. Differences between 2 not normally distributed populations have been tested for significance with the 2-tailed Mann–Whitney and Wilcoxon signed-rank tests for unpaired and paired data sets, respectively (95% confidence level). Categorical variables were compared using chi-square analyses or the Fisher exact test, as appropriate to the cell frequencies. All statistical calculations have been performed with the SPSS 16.0.2 program (SPSS Inc., Chicago, IL).

### Calculation of Lifetime Attributable Risk (LAR)

This study was not specifically designed for risk estimation. Nevertheless, indicative assessments of the lifetime attributable risk (LAR) for cancer incidence and mortality due to exposure during the interventional procedure were performed using two distinct approaches, to facilitate comparison with corresponding results in the literature. Estimates of sample patient effective dose were combined with sex and age specific LAR estimates per unit dose from the BEIR VII report (26) under the assumption of the linear-no threshold (LNT) model. Additionally, the above mentioned sex and age specific LAR estimates per unit dose were weighted by the relative number of γ-H2AX foci per cell induced by the interventional procedure and the *ex vitro* irradiation for each sample patient.

## Results

Baseline patients' demographics in the overall cohort are shown in Supplementary Table 1. The dicentric analysis was performed in 24 out of 25 patients, immunofluorescence analysis (γ-H2AX foci) in 22 out of 25 patients, whereas the CBMN assay was performed in 20 out of 25 patients. The mean effective dose to the 25 patients was 14.33 ± 12.8 mSv (median 11.2 mSv, range 1.74–52.52 mSv). The mean value of BMI was 29.2 ± 4.8 kg/m^2^ and the mean age was 63 ± 13 years.

**Figure 1:**
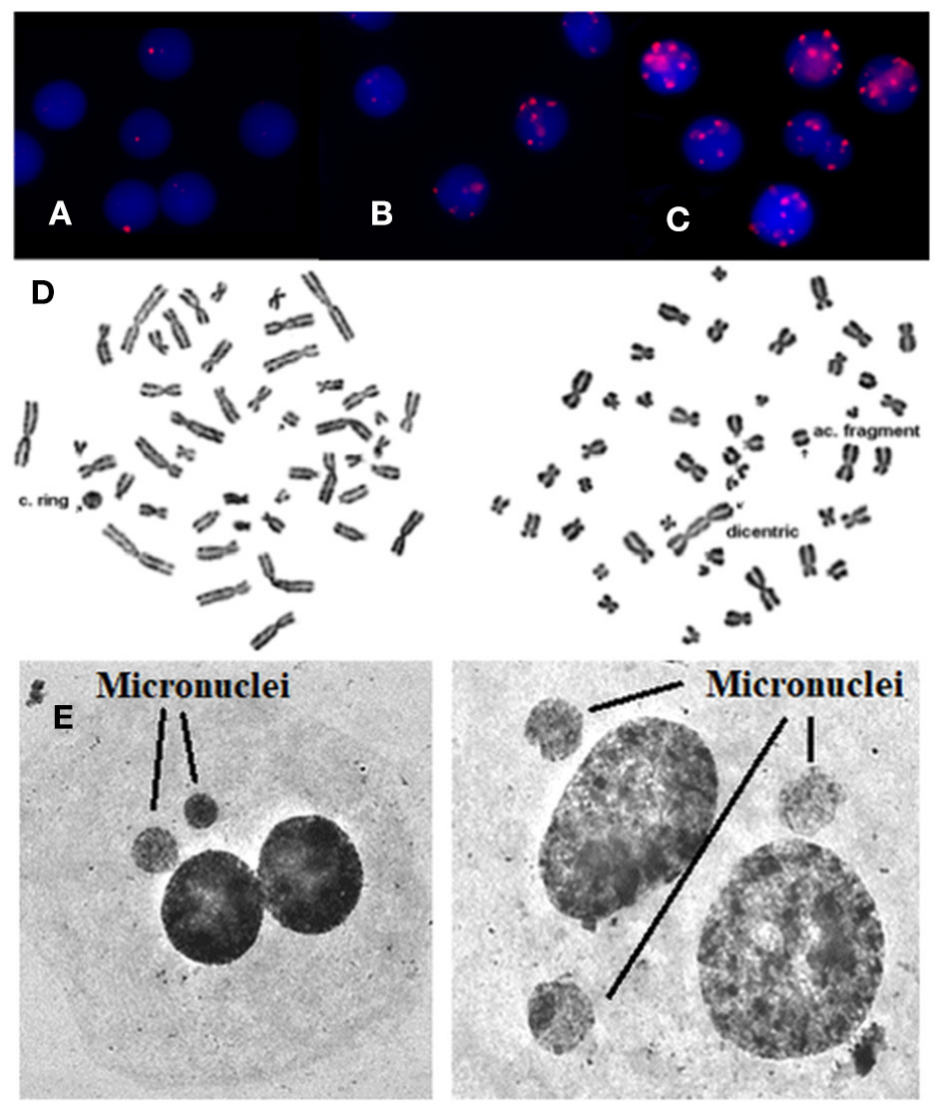
γ-H2AX foci are visualized by means of immunofluorescence staining. **(A)** Before the exposure, **(B)** after *in vivo* exposure, **(C)** after *in vitro* irradiation with 1 Gy, **(D)** using Giemsa staining, rings, and dicentric chromosomes can easily and accurately be detected and quantified in patients' blood samples. Arrows indicate chromosome aberrations. On the left there is a centric ring with acentric fragment and on the right there is a dicentric chromosome with acentric fragment, **(E)** using Giemsa staining: micronuclei can easily and accurately be detected and quantified in patients. Arrows indicate micronuclei.

**Figure 2:**
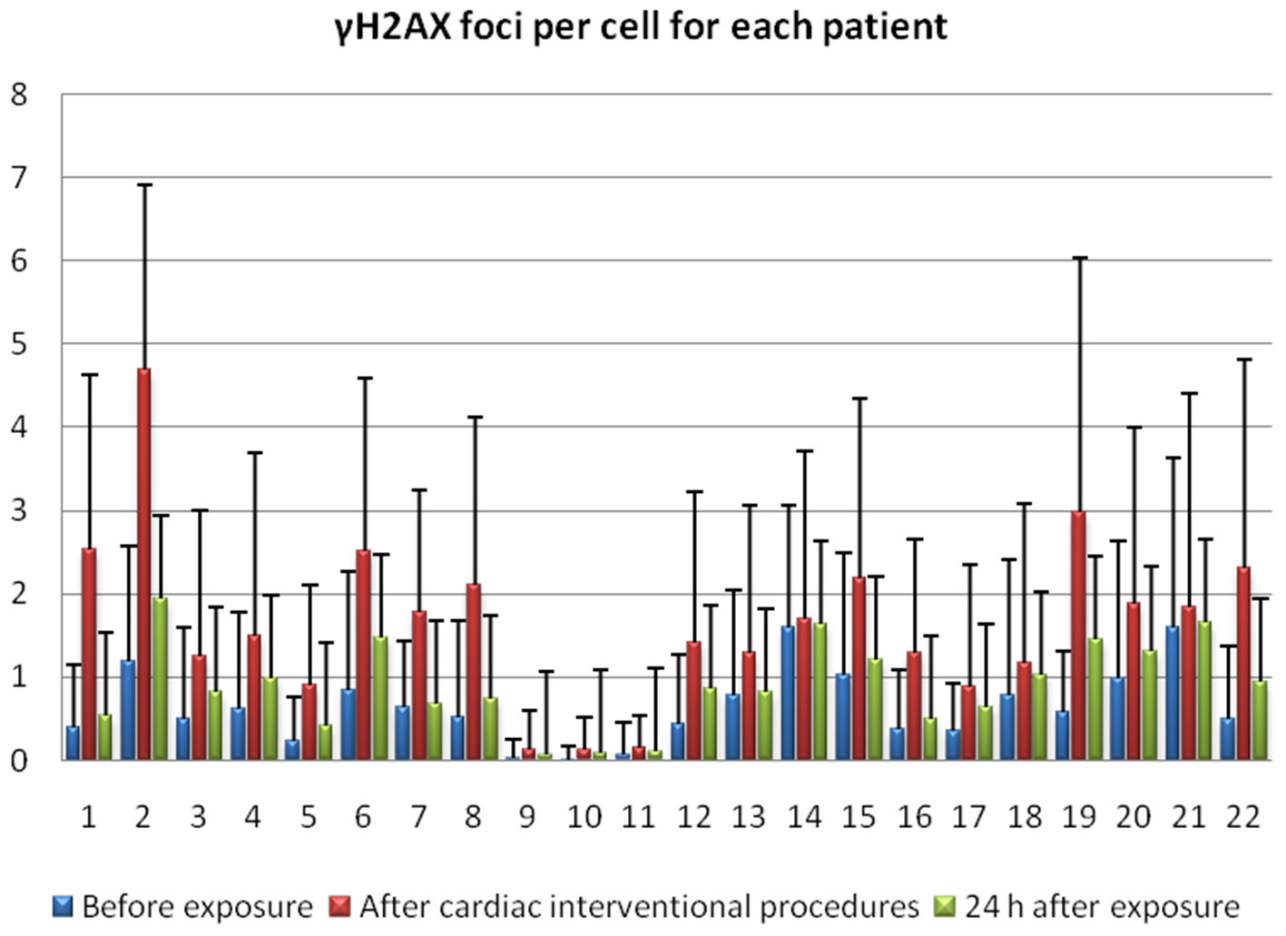
γ-H2AX foci for 22 individuals before the exposure, immediately after interventional cardiology procedure and 24 h following exposure.

The exposure even to low doses of IR (<100 mSv) seems to have biological effects on DNA. Particularly, with the immunofluorescence analysis, we observed significantly higher γ-H2AX foci frequencies (Figures 1A–C) after the interventional procedures and after *in vitro* irradiation compared to baseline (γ-H2AX foci frequencies before cardiac interventional procedures) (from 0.64 ± 0.43 at baseline to 1.66 ± 1.03 and 11.59 ± 0.89 for *in vivo* and *in vitro* 1 Gy exposures, respectively, *p* < 0.001 by paired *t*-tests, Tables 1, 2). All patients presented higher γ-H2AX foci after the interventional cardiac procedures and in the 91% of them the increase was statistically significant compared to baseline (*p* < 0.001 by paired *t*-tests, Figure 2). However, the number of γ-H2AX foci declined after 24 h but rarely reached the baseline level, irrespective if the exposure was at low or at high doses (0.91 ± 0.52 and 2.15 ± 1.10 for 24 h after *in vivo* and *in vitro* exposures, respectively, *p* < 0.001 compared to baseline, Tables 1, 2). After reaching their maximum value, the percentage of γ-H2AX foci that were repaired 24 h after the cardiac procedure varied between 25% and 95.6% (Table 1). The percentage of the remaining foci 24 h after *in vivo* exposure for the whole study group was 33.5% of their maximum value, whereas the percentage of the remaining foci after *in vitro* exposure was 13.7%. Finally, it was found that there is a correlation between the frequency of γ-H2AX foci and the fluoroscopy time (*r* = 0.520 and *p* = 0.013, by regression analysis) as well as a positive correlation between the frequency of γ-H2AX and the effective dose delivered to the patients (0.540 and *p* = 0.010, by regression analysis).

**Table 1:** Mean values of chromosomal aberrations, frequency of Micronuclei (MN), and γ-H2AX foci per cell before the exposure, immediately after the interventional procedure (*in vivo* radiation), and 24 h later, as well as the percentage of γ-H2AX foci repaired at 24 h.

**Patients**	**Chr. aberrations before the exposure**	**Chr. aberrations after the procedure**	**Frequency of MN before the exposure**	**Frequency of MN after the procedure**	**γ-H2AX foci before the exposure**	**γ-H2AX foci after the procedure**	**γ-H2AX foci 24 h after the procedure**	**Percentage of γ-H2AX foci repaired in 24 h**
1	0.001	0.002	0.018 ± 0.147	0.024 ± 0.153	–	–	–	–
2	0	0.003	0.007 ± 0.083	0.016 ± 0.09	0.39 ± 0.76	2.53 ± 2.12	0.54 ± 0.83	92.9%
3	0.003	0.003	0.005 ± 0.070	0.007 ± 0.083	1.19 ± 1.69	4.7 ± 2.21	1.94 ± 2.29	78.6%
4	0.003	0.004	0.006 ± 0.077	0.012 ± 0.108	0.50 ± 1.09	1.25 ± 1.75	0.83 ± 1.34	56%
5	0	0.003	0.01 ± 0.099	0.013 ± 0.11	0.62 ± 1.16	1.5 ± 2.19	0.98 ± 1.31	59.09%
6	0	0	0.005 ± 0.07	0.010 ± 0.09	0.23 ± 0.54	0.9 ± 1.21	0.41 ± 0.69	73.1%
7	0.001	0.004	0.009 ± 0.09	0.016 ± 0.125	0.85 ± 1.41	1.26 ± 1.51	1.03 ± 1.42	62.04%
8	0	0	0.003 ± 0.05	0.007 ± 0.08	0.63 ± 0.81	1.78 ± 1.47	0.68 ± 1.01	95.6%
9	0	0	–	–	0.52 ± 1.16	2.1 ± 2.03	0.74 ± 1.02	86%
10	0	0	–	–	0.03 ± 0.23	0.12 ± 0.483	0.07 ± 0.25	55.5%
11	0	0.003	–	–	0.01 ± 0.16	0.12 ± 0.4	0.08 ± 0.27	36.3%
12	0	0.002	–	–	0.07 ± 0.38	0.15 ± 0.39	0.13 ± 0.33	25%
13	–	–	–	–	0.44 ± 0.83	1.42 ± 1.81	0.87 ± 1.09	56.1%
14	0	0.001	0.007 ± 0.083	0.014 ± 0.117	0.78 ± 1.26	1.29 ± 1.78	0.82 ± 1.09	92.1%
15	0.001	0.001	0.005 ± 0.07	0.04 ± 0.20	1.59 ± 1.48	1.69 ± 2.03	1.63 ± 1.78	60%
16	0	0	0.007 ± 0.08	0.066 ± 0.24	1.02 ± 1.48	2.18 ± 2.16	1.2 ± 1.8	84.4%
17	0	0.001	0.006 ± 0.077	0.019 ± 0.136	0.38 ± 0.70	1.3 ± 1.35	0.65 ± 0.96	70.6%
18	0	0.001	0.009 ± 0.094	0.011 ± 0.104	0.35 ± 0.57	0.88 ± 1.46	0.64 ± 1.02	45.2%
19	0	0.002	0.003 ± 0.054	0.028 ± 0.165	–	–	–	–
20	0	0.002	0.009 ± 0.09	0.017 ± 0.129	0.79 ± 1.62	1.16 ± 1.92	1.03 ± 1.31	35.1%
21	0	0.002	0.009 ± 0.094	0.013 ± 0.113	0.58 ± 0.73	0.95 ± 1.45	1.46 ± 1.78	63.4%
22	0	0.001	0.007 ± 0.083	0.016 ± 0.125	0.98 ± 1.66	1.89 ± 2.1	1.32 ± 1.7	62.6%
23	0	0.002	0.008 ± 0.089	0.023 ± 0.15	1.59 ± 2.04	1.85 ± 2.55	1.66 ± 1.75	73%
24	0	0.002	0.005 ± 0.070	0.013 ± 0.113	0.49 ± 0.88	2.31 ± 2.51	0.94 ± 1.23	75.2%
25	0	0.001	0.008 ± 0.089	0.042 ± 0.20	–	–	–	–
Mean value	0.00038 ± 0.000875	0.00163 ± 0.00128	0.0073 ± 0.0032	0.0204 ± 0.0143	0.64 ± 0.43	1.66 ± 1.03	0.91 ± 0.52	

**Table 2:** Mean values of γ-H2AX foci and frequency of Micronuclei (MN) per cell after cardiac interventional procedures (*in vivo* radiation) and after irradiation with 1 Gy in the laboratory (*in vitro* radiation).

**Patients**	**Frequency of MN after the procedure**	**Frequency of MN after irradiation with 1 Gy**	**γ-H2AX foci after the procedure**	**γ-H2AX foci after irradiation with 1 Gy**
1	0.024 ± 0.153	–	–	–
2	0.016 ± 0.09	0.03 ± 0.19	2.53 ± 2.09	12.6 ± 3.11
3	0.007 ± 0.083	0.052 ± 0.23	4.7 ± 2.21	13.4 ± 2.38
4	0.012 ± 0.108	0.08 ± 0.28	2.65 ± 2.31	12.9 ± 2.41
5	0.013 ± 0.11	0.065 ± 0.26	1.5 ± 2.19	10.9 ± 2.48
6	0.010 ± 0.09	0.060 ± 0.237	2.01 ± 3.18	10.9 ± 2.48
7	0.016 ± 0.125	0.064 ± 0.248	2.51 ± 2.08	10.9 ± 2.39
8	0.007 ± 0.08	0.057 ± 0.25	1.78 ± 1.47	11.9 ± 2.45
9	–	–	1.28 ± 1.6	12.6 ± 2.39
10	–	–	0.12 ± 0.483	10.6 ± 2.31
11	–	–	0.12 ± 0.4	11.7 ± 2.5
12	–	–	0.15 ± 0.39	10.6 ± 2.3
13	–	–	1.42 ± 1.81	11.01 ± 2.1
14	0.014 ± 0.117	–	1.29 ± 1.78	11.03 ± 2.17
15	0.04 ± 0.20	0.23 ± 0.54	1.69 ± 2.03	11.4 ± 3.23
16	0.066 ± 0.24	0.18 ± 0.38	2.18 ± 2.16	10.7 ± 2.48
17	0.019 ± 0.136	0.114 ± 0.33	0.86 ± 1.13	10.6 ± 4.43
18	0.011 ± 0.104	0.058 ± 0.233	1.38 ± 1.84	12.7 ± 2.42
19	0.028 ± 0.165	0.114 ± 0.317	–	–
20	0.017 ± 0.129	0.064 ± 0.244	1.16 ± 1.92	11.3 ± 2.44
21	0.013 ± 0.113	0.066 ± 0.248	2.99 ± 3.04	13.02 ± 2.33
22	0.016 ± 0.125	0.12 ± 0.325	1.89 ± 2.1	10.9 ± 2.31
23	0.023 ± 0.15	0.061 ± 0.239	1.85 ± 2.55	11.8 ± 2.49
24	0.013 ± 0.113	0.1 ± 0.31	2.31 ± 2.51	11.6 ± 2.58
25	0.042 ± 0.20	0.085 ± 0.279	–	–
Mean value	0.0204 ± 0.0143	0.089 ± 0.049	1.66 ± 1.03	11.59 ± 0.89

Regarding the dicentric analysis and based on 1,000 analyzed cells per experimental point, we observed significantly higher frequencies of dicentric chromosomes and centric rings for the total study group after the exposure compared to the baseline (Table 1). The baseline mean value of chromosomal aberrations was 0.00038 ± 0.000875 per cell and the yield increased to 0.00163 ± 0.00128 (*p* < 0.001 by Wilcoxon signed-rank test) following interventional cardiac procedures and exposure to low doses. In contrary, no significant increase in chromosomal aberrations frequency was observed at the individual patient level (*p* > 0.05 by Wilcoxon signed-rank test). Images from chromosomal aberrations are shown in Figure 1D.

Using the CBMN assay, the number of micronuclei in 1,000 binucleated lymphocytes after the interventional procedures were significantly increased (*p* < 0.001 Wilcoxon signed-rank test) for the total study at the group level (0.0204 ± 0.0143) as compared to the cells analyzed before medical exposure (0.0073 ± 0.0032) (Table 1). Only in 25% of the donors showed a statistical significant increase in the number of micronuclei after the interventional cardiac procedures. Images from micronuclei are shown in Figure 1E.

Finally, concerning the calculation of the lifetime attributable risk, under the assumption of the LNT model, the median LAR for total cancer incidence and mortality in the patient sample was found equal to 0.046% (range: 0.005–0.291) and 0.032% (range: 0.004–0.179), respectively. Corresponding results from γ-H2AX foci measurements were 0.625% (range: 0.051–1.999) and 0.423% (range: 0.036–1.354).

## Discussion

In the present study, we used biomarkers such as γ-H2AX foci and formation of dicentric chromosomes and micronuclei (MN), which underpin radiation-induced DNA damage, to assess the potency and the suitability of these genotoxicity endpoints to detect and quantitate biological effects in peripheral blood lymphocytes of 25 patients undergoing interventional cardiology treatment. Even though the mean values of all patients as a group for all three endpoints tested show increased yields relative to baseline following medical exposure, our results demonstrate that only the γ-H2AX biomarker enables detection of statistically significant differences at the individual level (*p* < 0.001) for almost all patients (91%).

The present observations agree with cytogenetic studies carried out in patients exposed to low IR doses. Particularly, according some studies the numbers of dicentric and ring chromosomes were significantly increased after a CT scan in patients (13, 19, 27–29). In our study, after the cardiac interventional procedures, which have resulted in a mean dose of about 14 mSv a total of 48.000 cells have been analyzed. The mean frequency of chromosomal aberrations caused by cardiac interventional procedures is 0.00163 ± 0.00128 per cell. However, despite the statistically significant increase in the frequency of chromosomal aberrations as compared to the baseline of the whole group of patients, no significantly higher frequency of dicentrics was observed for each patient individually after the interventional procedures.

Moreover, several reports noted significantly higher numbers of micronuclei after exposure to low doses of IR (13, 29). Our results are in agreement with these findings implying that even low ionizing radiation doses may cause a higher incidence of MN. Specifically, in our study our recorded values of effective doses for these catheterization procedures ranged between 2–52 mSv and there was a significant higher frequency in MN for the total study group reaching the mean value of 0.0204 ± 0.0143, as compared with the baseline (0.0073 ± 0.0032) per cell. Nevertheless, at the individual level, only in 25% of them the increase was statistically significant. The micronucleus assay in human lymphocytes is one of the most commonly used method for measuring DNA damage (20) but it is considered to have a less sensitivity compared to the dicentric analysis (30). On the other side, the key advantage of the micronucleus assay lies in its ability to detect both clastogenic and aneugenic events such as asymmetrical cell divisions, which may partially explain our results. The disadvantage of the CBMN assay is related to the variable micronucleus background frequency, and this is an important limitation in these studies (31). However, MN and chromosomal aberrations are considered to be cellular biomarkers of chromosome damage and early predictors of increased cancer risk (32). Indeed, radiation-induced MN may contribute to genomic instability through chromosome shattering and chromothripsis within MN caused by premature chromosome condensation in case of asynchronous cell-cycle progression between main and MN (33). Also, according to Vral et al., the conventional CBMN assay is a thoroughly validated and standardized technique in the field of radiation biology as it can be used to evaluate *in vivo* radiation exposure of occupational, medical, and accidentally exposed individuals and to assess individual *in vitro* radiosensitivity or cancer susceptibility. Radiation-induced chromosome aberrations such as MN are mainly the result of unrepaired or misrepaired DSBs (31).

At a molecular level, there are certain studies that report an increase of DSBs after exposure to low ionizing radiation doses. Kuefner et al. (34) and Alipoor et al. (35) observed an increase in the percentage of DSBs after exposure to angiography, compared to pre-exposure time. Similarly, in our study, not only a statistically significant increase in the mean value of DNA DSBs was observed for the total study group, but also at the individual level, this increase was statistically significant in the 91% of patients after their cardiac interventional procedures and residual γ-H2AX foci were still detectable in irradiated lymphocytes, 24 h after.

Among the three biomarkers used, the γ-H2AX foci assay, demonstrated a positive correlation between the frequency of γ-H2AX foci and the fluoroscopy time (*r* = 0.520 and *p* = 0.013) and a linear positive correlation between the frequency of γ-H2AX foci and effective dose (*r* = 0.540 and *p* = 0.010). This result is in accordance with earlier studies that reported a positive correlation in patients exposed to low radiation doses (35, 36).

Furthermore, Geisel et al. (37) found that 1 day (24 h) after irradiation there was a complete repair of DSBs (as visualized by γ-H2AX foci) to background levels, whereas Grudzenski et al. found that barely any foci loss was observed after 24 h after 10 mGy (38). These results are in agreement with several studies which showed a higher significant foci mean value 24 h following irradiation compared to the pre-irradiation baseline mean value (38, 39). However, the decline in the γ-H2AX foci values 24 h after irradiation varied significantly among the different individuals, ranging from 25 to 95.6%.These results indicate a great variability in the repair kinetics of γ-H2AX foci.

The repair kinetics of γ-H2AX foci are complex and depend on many factors (36). Studies indicate that the rate of foci loss and presence of residual foci has been correlated with cellular radiosensitivity (40). One cannot exclude the possibility of *de novo* generation of DSBs due to the processing (repair) of clustered DNA lesions even at such low doses (41).

It has become apparent over recent years that there is a variability in the radiation sensitivity among different individuals in the population (42). The detrimental effects of IR on DNA are well-known and profound, however, the dependence of radiosensitivity on the repair capacity is also explored to clarify the open questions in radiobiology which may be very crucial at the level of low doses. The DNA damage induced after low dose exposure in combination with a possible increased intrinsic radiosensitivity, may underlie the increased level of γ-H2AX foci in individuals 24 h following the interventional cardiac procedures.

It has also been reported by Grudzenski et al. that the kinetics of γ-H2AX foci loss are strongly dependent on dose, with cells exposed to 200 mGy or higher showing much faster repair kinetics than cells irradiated with a few milligray of X-rays (38). Furthermore, a recent study from Jakl et al. indicated that human lymphocytes seem to be more sensitive to low doses ( ≤ 10 cGy) as compared to higher doses as ionizing radiation-induced foci (IRIF) induced in human lymphocytes by low doses persist longer (43). It is important though to be mentioned that in our study, our experiments were not performed at 72 h post-irradiation time point but up to 24 h and that was accomplished into the laboratory *in vitro*. So, we didn't receive blood samples from each patient 24 h after their medical treatment to investigate the repair kinetics in an *in vivo* way. The *in vitro* repair of DSBs may be less efficient than it could be the after *in vivo* repair. According to Belyaev it is more likely that residual foci represent some unprepared changes in chromatin conformation (44) so, some of complex DSB require longer time to be repaired. In this way formation of chromosomal exchanges may be produced (44).

Moreover, the fate of the misrepaired radiation-induced DSBs still remains to be elucidated and the potential ability of chromosome aberrations to induce asymmetric cell division, micronuclei, and chromosomal instability according to the intrinsic radiosensitivity of each individual. Erroneous repair of DNA DSBs can result in chromosomal rearrangements which are associated with tumorigenesis. An increase in chromosomal aberrations as it happens in ataxia-telangiectasia patients, leads to genetic instability which enhances the rate of cancer development (45). Even though many studies point toward a link between carcinogenesis and exposure to radiation, the exact mechanism is still not clear. Induction of genomic instability is suspected to play a major role in malignant transformation after high-dose irradiation, and it might be responsible for potential carcinogenesis after exposure to lower doses (46).

Finally, results of γ-H2AX foci induction per cell of this work also suggest a considerably greater LAR from the interventional procedure in the patient sample relative to that estimated under the LNT model assumption. This is in agreement with findings of Beels et al. (47) reporting mortality risk estimates based on γ-H2AX foci much higher than those expected from the LNT model in a sample of pediatric patients who underwent cardiac catheterization procedures. The remarkable difference in the magnitude of this increase [~4-fold in Beels et al. (47) relative to 13-fold in this work] is explained, in part, by the use of ^60^Co for the *in vitro* irradiation in this work, since the risk from exposure to x-rays could be greater than that for γ rays by a factor of 2 or 3 (26).

The estimation of the radiation risk of low-dose radiation ( ≤ 100 mSv), remains challenging and our study is in line with Beels et al. (47). Especially, according to Raavi et al. (48) measurement of γ-H2AX foci is a rapid and sensitive method that does not require culturing and thus it can be used as a potential marker to study the levels of DNA damage after CT procedures (48). CBMN assay has limitations on using as a biological dosimeter particularly for low doses due to the great variability (49).

## Conclusion

From the three different endpoints investigated in the present study the γ-H2AX foci could function as biomarker of exposure after interventional cardiac procedures displaying low dose radiation effects. In addition, the number of γ-H2AX foci declined 24 h following exposure, but rarely reached the baseline level, irrespective of the radiation dose, illustrating variability in the kinetics of the γ-H2AX foci among the different individuals. Furthermore, in this study, it is clearly being proved that the immunofluorescence assay is a very sensitive method to detect DNA damage after exposure to very low doses as the increase was statistically significant in the 91% of patients studied.

Regarding the cytogenetic and MN data obtained, an increase in the frequency of chromosomal aberrations and MN after the interventional procedures was observed for the whole study group but not at the individual level for the dicentrics, whereas only a 25% of the whole group studied showed a significant increase in the MN. These observations point up the clear advantage of the use of γ-H2AX foci over the conventional dicentric and micronuclei assays for low level doses. Finally, results of γ-H2AX foci induction per cell of this work also show a considerably greater LAR from the interventional procedure in the patient sample relative to that estimated under the LNT model assumption. Thus, more radiobiological research is needed and a better understanding of the molecular mechanisms involved would be crucial for low-dose exposure risk estimation for radiation workers, patients, and people exposed to high background radiation.

## Study Limitations

An undoubted limitation of our study is that patient's follow-up is not available. This leads unavoidably to a true difficulty in delineating the risk of the biological effects and investigating the repair mechanisms after exposure to low-doses as the samples from patients could not be repeatedly obtained.

MN assay also has some limitations that need to be acknowledged. The value of MN frequency as a long-term predictor of cancer was recently established, but more confirmatory data, which means more patients are certainly needed at this point. In addition, a wide range of clastogenic and aneugenic agents (i.e., agents causing chromosome breakages and abnormal number of chromosomes, respectively) can induce MN.

## Data Availability Statement

The original contributions presented in the study are included in the article/Supplementary Material, further inquiries can be directed to the corresponding author/s.

## Ethics Statement

The studies involving human participants were reviewed and approved by Ethics Committee of UoA (University of Athens). The patients/participants provided their written informed consent to participate in this study.

## Author Contributions

MH designed the study, wrote the first draft of the manuscript, and performed the investigation. PKK wrote sections of the manuscript, organized the database, and performed the statistical analysis. AN contributed to the investigation. PP and PK reviewed the manuscript. AG contributed to conception and design of the study. VH contributed to methodology and investigation. IMal, NK, and IMas contributed to the organization of the database. VV responsible for patients' database and reviewed the manuscript. GT supervised and critical reviewed the manuscript. All authors contributed to the article and approved the submitted version.

## Conflict of Interest

The authors declare that the research was conducted in the absence of any commercial or financial relationships that could be construed as a potential conflict of interest.

## Publisher's Note

All claims expressed in this article are solely those of the authors and do not necessarily represent those of their affiliated organizations, or those of the publisher, the editors and the reviewers. Any product that may be evaluated in this article, or claim that may be made by its manufacturer, is not guaranteed or endorsed by the publisher.

## References

[1] Picano E Andreassi MG Piccaluga E Cremonesi A Guagliumi G. Occupational risks of chronic low dose radiation exposure in cardiac catheterisation laboratory: the Italian Healthy Cath Lab Study. EMJ Int Cardiol. (2013) 1:50–8.

[2] Ho TL Shieh SH Lin CL Shen WC Kao CH. Risk of cancer among cardiologists who frequently perform percutaneous coronary interventions: a population-based study. Eur J Clin Invest. (2016) 46:527–34. 10.1111/eci.12628 27018993

[3] National Council on Radiation Protection and Measurements. NCRP Report No. 160 (2009). p. 242–3.

[4] Picano E Vañó E Rehani MM Cuocolo A Mont L Bodi V. The appropriate and justified use of medical radiation in cardiovascular imaging: a position document of the ESC Associations of cardiovascular imaging, percutaneous cardiovascular interventions and electrophysiology. Eur Heart J. (2014) 35:665–72. 10.1093/eurheartj/eht394 24401558

[5] Chen J Einstein AJ Fazel R Krumholz HM Wang Y Ross JS. Cumulative exposure to ionizing radiation from diagnostic and therapeutic cardiac imaging procedures: a population-based analysis. J Am Coll Cardiol. (2010) 56:702–11. 10.1016/j.jacc.2010.05.014 20619569 PMC2952402

[6] Mathews JD Forsythe AV Brady Z Butler MW Goergen SK Byrnes GB. Cancer risk in 680,000 people exposed to computed tomography scans in childhood or adolescence: data linkage study of 11 million Australians. BMJ. (2013) 346:f2360. 10.1136/bmj.f2360 23694687 PMC3660619

[7] Bethesda MD. Uncertainties in the Estimation of Radiation Risks and Probability of Disease Causation. National Council on Radiation Protection and Measurements (NCRP). NCRP Report No. 171 (2012). p. 418.

[8] Kitahara CM Linet MS Rajaraman P Ntowe E Berrington de González A. A new era of low-dose radiation epidemiology. Curr Envir Health Rep. (2015). 2:236–49. 10.1007/s40572-015-0055-y 26231501 PMC10267798

[9] Asaithamby A Chen DJ. Cellular responses to DNA double-strand breaks after low-dose γ-irradiation. Nucleic Acid Res. (2009) 37:3912–23. 10.1093/nar/gkp237 19401436 PMC2709554

[10] Rasnick D. Chapter 5: The chromosomal imbalance theory of cancer. In: The Autocatalyzed Progression of Aneuploidy is Carcinogenesis. 1st ed. Boca Raton, FL: CRC Press Taylor and Francis Group (2011). p. 330. 10.1201/b11665

[11] Mariotti LG Pirovano G Savage KI Ghita M Ottolenghi A Prise KM. Use of the γ-H2AX assay to investigate dna repair dynamics following multiple radiation exposures. PLoS ONE. (2013) 8:e79541. 10.1371/journal.pone.0079541 24312182 PMC3843657

[12] Rogakou EP Papadakis V Chrousos GP. The Epigenetic Biomarker γH2AX: From Bench to Clinical Trials. In: Hollar D, editor. Epigenetics, the Environment, and Children's Health Across Lifespan. Cham: Springer (2016). p. 93–115. 10.1007/978-3-319-25325-1_4

[13] Shi L Tashiro S. Estimation of the effects of medical diagnostic radiation exposure based on DNA damage. J Radiat Res. (2018) 59:ii121–9. 10.1093/jrr/rry006 29518207 PMC5941141

[14] Moquet J Bernard S Staynova A Lindholm C Gil OM Martins V. The second gamma-H2AX assay inter-comparison exercise carried out in the framework of the European biodosimetry network (RENEB). Int J Radiat Biol. (2017) 93:58–64. 10.1080/09553002.2016.1207822 27686523

[15] Leonard A Rueff J Gerber GB Leonard ED. Usefulness and limits of biological dosimetry based on cytogenetic methods. Radiat Prot Dosim. (2005) 115:448–54. 10.1093/rpd/nci061 16381765

[16] Qian Q-Z Cao X-K Shen F-H Wang Q. Effects of ionising radiation on micronucleus formation and chromosomal aberrations in Chinese radiation workers. Radiat Prot Dosimetry. (2016). 168:197–203. 10.1093/rpd/ncv290 26084304 PMC4884887

[17] Durante M., Formenti SC. Radiation-induced chromosomal aberrations and immunotherapy: micronuclei, cytosolic, DNA, and interferon-production pathway. Front Oncol. (2018) 8:192. 10.3389/fonc.2018.00192 29911071 PMC5992419

[18] Kulka U Abend M Ainsbury E Badie C Barquinero JF Barrios L. RENEB - Running the European Network of biological dosimetry and physical retrospective dosimetry. Int J Radiat Biol. (2017) 93:2–14. 10.1080/09553002.2016.1230239 27707245

[19] Stephan G Schneider K Panzer W Walsh L Oestreicher U. Enhanced yield of chromosome aberrations after CT examinations in paediatric patients. Int J Radiat Biol. (2007) 83:281–7. 10.1080/09553000701283816 17457753

[20] Fenech M. Biomarkers of genetic damage for cancer epidemiology. Toxicology. (2002) 181–182:411–6. 10.1016/S0300-483X(02)00480-8 12505344

[21] Saha J Wang SY Davis AJ. Examining DNA double-strand break repair in a cell cycle-dependent manner. Methods Enzymol. (2017) 591:97–118. 10.1016/bs.mie.2017.03.012 28645381 PMC5580029

[22] Pantelias A Zafiropoulos D Cherubini R Sarchiapone L De Nadal V Pantelias EG. Interphase cytogenetic analysis of Go lymphocytes exposed to α-particles, C-ions, and protons reveals their enhanced effectiveness for localized chromosome shattering- a critical risk for chromothripsis. Cancers. (2020) 12:2336. 10.3390/cancers12092336 PMC7563219 32825012

[23] Fenech M. Cytokinesis-block micronucleus cytome assay. Nature Protocols. (2007) 2:1084–104. 10.1038/nprot.2007.77 17546000

[24] Kocinaj D Cioppa A Ambrosiani G Tesorio T Salemme L Sorropago G. Radiation dose exposure during cardiac and peripheral arteries catheterisation. Int J Cardiol. (2006) 113:283–4. 10.1016/j.ijcard.2005.09.035 16330116

[25] Simantirakis G Koukorava C Kalathaki M Pafilis C Kaisas I Economides S. Reference levels and patient doses in interventional cardiology procedures in Greece. Eur Radiol. (2013) 23:2324–32. 10.1007/s00330-013-2813-2 23559142

[26] BEIR. Health Risks from exposure to low levels of ionizing radiation: BEIR VII. Washington, DC: National Academic Press (2006). 25077203

[27] Shi L Fujioka K Sakurai-Ozato N Fukumoto W Satoh K Sun J. Chromosomal abnormalities in human lymphocytes after computed tomography scan procedure. Radiat Res. (2018) 190:424–32. 10.1667/RR14976.1 30040044

[28] Abe Y Miura T Yoshida MA Ujiie R Kurosu Y Kato N. Analysis of chromosome translocation frequency after a single CT scan in adults. J Radiat Res. (2016) 57:220–6. 10.1093/jrr/rrv090 26874116 PMC4915535

[29] Kanagaraj K Abdul Syed Basheerudeen S Tamizh Selvan G Jose MT Ozhimuthu A Selvam SP. Assessment of dose and DNA damages in individuals exposed to low dose and low dose rate ionizing radiations during computed tomography imaging. Mutat Res Genet Toxicol Environ Mutagen. (2015) 789–790:1–6. 10.1016/j.mrgentox.2015.05.008 26232253

[30] Hayashi M. The micronucleus test- most widely used *in vivo* genotoxicity test. Genes Environ. (2016) 38:18. 10.1186/s41021-016-0044-x 27733885 PMC5045625

[31] Vral A Fenech M Thierens H. The micronucleus assay as a biological dosimeter of *in vivo* ionising radiation exposure. Mutagenesis. (2011) 26:11–7. 10.1093/mutage/geq078 21164177

[32] Andreassi MG Cioppa A Manfredi S Palmieri C Botto N Picano E. Acute chromosomal DNA damage in human lymphocytes after radiation exposure in invasive cardiovascular procedures. Eur Heart J. (2007) 28:2195–9. 10.1093/eurheartj/ehm225 17598926

[33] Pantelias A Karachristou I Georgakilas A Terzoudi G. Interphase cytogenetic analysis of micronucleated and multinucleated cells supports the premature chromosome condensation hypothesis as the mechanistic origin of chromothripsis. Cancers. (2019) 11:1123. 10.3390/cancers11081123 31390832 PMC6721583

[34] Kuefner MA Brand M Engert C Schwab SA Uder M. Radiation induced DNA double-strand breaks in radiology. Rofo. (2015) 187:872–8. 10.1055/s-0035-1553209 26333102

[35] Alipoor A Fardid R Sharifzadeh S. Evaluating gamma-H2AX expression as a biomarker of DNA damage after x-ray in angiography patients. J Biomed Phys Eng. (2018) 8:393–402. 10.31661/jbpe.v8i4Dec.768 30568929 PMC6280120

[36] Halm BM Franke AA Lai JF Turner HC Brenner DJ Zohrabian VM. γ-H2AX foci are increased in lymphocytes *in vivo* in young children 1 h after very low-dose X-irradiation: a pilot study. Pediatr Radiol. (2014) 44:1310–7. 10.1007/s00247-014-2983-3 24756254 PMC4175172

[37] Geisel D Zimmermann E Rief M Greupner J Laule M Knebel F. DNA double-strand breaks as potential indicators for the biological effects of ionising radiation exposure from cardiac CT and conventional coronary angiography: a randomised, controlled study. Eur Radiol. (2012) 22:1641–50. 10.1007/s00330-012-2426-1 22527372

[38] Grudzenski S Raths A Conrad S Rube C Lobrich M. Inducible response required for repair of low-dose radiation damage in human fibroblasts. Proc Natl Acad Sci USA. (2010) 107:14205–10. 10.1073/pnas.1002213107 20660770 PMC2922519

[39] Nair S Engelbrecht M Miles X Ndimba R Fisher R Plessis P. The impact of dose rate on DNA double-strand break formation and repair in human lymphocytes exposed to fast neutron irradiation. Int J Mol Sci. (2019) 20:5350. 10.3390/ijms20215350 31661782 PMC6862539

[40] Sharma PM Ponnaiya B Taveras M Shuryak I Turner H Brenner DJ. High throughput measurement of γ-H2AX DSB repair kinetics in a healthy human population. PLoS ONE. (2015) 10:e0121083. 10.1371/journal.pone.0121083 25794041 PMC4368624

[41] Georgakilas AG O'Neill P Stewart RD. Induction and repair of clustered DNA lesions: what do we know so far? Radiat Res. (2013) 180:100–9. 10.1667/RR3041.1 23682596

[42] Bouffler SD. Evidence for variation in human radiosensitivity and its potential impact on radiological protection. Ann ICRP. (2016) 45(Suppl. 1):280–9. 10.1177/0146645315623158 26956676

[43] Jakl L Markova E Kolarikova L Belyaev I. Biodosimetry of low-dose ionizing radiation using DNA repair foci in human lymphocytes. Genes. (2020) 11:58. 10.3390/genes11010058 31947954 PMC7016656

[44] Belyaev IY. Radiation-induced DNA repair foci: spatio-temporal aspects of formation, application for assessment of radiosensitivity and biological dosimetry. Mutation Res. (2010) 704:132–41 10.1016/j.mrrev.2010.01.011 20096808

[45] Alhmoud JF Woolley JF Moustafa AE Malki MI. DNA damage/repair management in cancers. Cancers. (2020) 12:1050. 10.3390/cancers12041050 32340362 PMC7226105

[46] Kulcenty K Suchorska WM Skrobała A Skorska M Kruszyna-Mochalska M Kowalik A. Carcinogenesis induced by low-dose radiation. Radiol Oncol. (2017) 51:369–77. 10.1515/raon-2017-0044 29333114 PMC5765312

[47] Beels L Bacher K Wolf D Werbrouck J Thierens H. γ-H2AX foci as a biomarker for patient x-ray exposure in pediatric cardiac catheterization. Are we underestimating radiation risks? Circulation. (2009) 120:1903–9. 10.1161/CIRCULATIONAHA.109.880385 19858412

[48] Raavi V Perumal V Paul FDS. Potential application of γ-H2AX as a biodosimetry tool for radiation triage. Mutat Res. (2021) 787:108350. 10.1016/j.mrrev.2020.108350 34083048

[49] International Atomic Energy Agency. Cytogenetic Analysis for Radiation Dose Assessment. Vienna: Technical reports series No. 405 (2001).

