# Supplementary material for: The Use of Genotoxicity Endpoints as Biomarkers of Low Dose Radiation Exposure in Interventional Cardiology
Source: Front Public Health. 2021 Jul 23;9:701878. doi: 10.3389/fpubh.2021.701878 (PMC8342993; doi:10.3389/fpubh.2021.701878)
Supplement: Supplementary file 1 [file Table_1.DOC]

**Table 1S.** Clinical and demographic characteristics of the study patients (CA: Coronary Angiography, PTCA: Percutaneous Transluminal Coronary Angioplasty)

| **Patients** | **Sex** | **Age** | **BMI** | **Procedure** | **DAP (Gy·cm^2^)** | **Effective Dose (mSv)** | **Fluoroscopy time (min)** | **Dicentric Analysis** | **Micronuclei Analysis** | **γ-Η2ΑΧ Foci** |
| --- | --- | --- | --- | --- | --- | --- | --- | --- | --- | --- |
| **1** | F | 66 | 22.30 | CA+PTCA | 21.88 | 5.688 | 11 |  |  | - |
| **2** | M | 44 | 32.30 | Ablation | 91.38 | 23.75 | 11.5 |  |  |  |
| **3** | M | 66 | 29.70 | Ablation | 202.00 | 52.52 | 59.9 |  |  |  |
| **4** | M | 62 | 30.10 | CA+PTCA | 20.60 | 5.36 | 6 |  |  |  |
| **5** | M | 52 | 37.70 | CA | 38.00 | 9.88 | 6 |  |  |  |
| **6** | M | 75 | 29.40 | CA | 6.71 | 1.74 | 0.6 |  |  |  |
| **7** | M | 80 | 24.50 | CA+PTCA | 52.30 | 13.59 | 16.4 |  |  |  |
| **8** | M | 56 | 31.00 | CA | 37.40 | 9.72 | 7.4 |  |  |  |
| **9** | F | 77 | 27.30 | CA+PTCA | 67.00 | 17.42 | 10.6 |  | - |  |
| **10** | M | 36 | 29.10 | Ablation | 43.90 | 11.41 | 5.1 |  | - |  |
| **11** | M | 69 | 26.80 | Ablation | 43.90 | 11.41 | 30 |  | - |  |
| **12** | M | 29 | 30.50 | CA+PTCA | 59.00 | 15.34 | 10.1 |  | - |  |
| **13** | M | 78 | 23.53 | CA+PTCA | 57.04 | 14.83 | 15.4 | - | - |  |
| **14** | M | 71 | 23.84 | CA | 12.26 | 3.19 | 2.6 |  |  |  |
| **15** | M | 72 | 35.01 | PTCA | 52.54 | 13.66 | 9.3 |  |  |  |
| **16** | M | 52 | 34.30 | CA+PTCA | 195.00 | 50.70 | 38.7 |  |  |  |
| **17** | M | 74 | 32.40 | CA+PTCA | 64.43 | 16.75 | 7.9 |  |  |  |
| **18** | F | 56 | 35.50 | CA | 14.24 | 3.70 | 3.2 |  |  |  |
| **19** | M | 64 | 26.40 | CA+PTCA | 55.91 | 14.54 | 9.7 |  |  |  |
| **20** | M | 52 | 26.30 | CA+PTCA | 77.78 | 20.22 | 6 |  |  |  |
| **21** | M | 61 | 24.20 | CA | 9.50 | 2.47 | 2.6 |  |  | - |
| **22** | M | 67 | 34.70 | CA | 13.53 | 3.52 | 4.7 |  |  |  |
| **23** | M | 82 | 20.60 | CA | 22.43 | 5.83 | 2.9 |  |  |  |
| **24** | M | 77 | 26.80 | CA+PTCA | 81.91 | 21.29 | 12.1 |  |  |  |
| **25** | M | 61 | 36.40 | CA | 39.45 | 10.26 | 14.6 |  |  | - |
